# Predicting Summer Site Occupancy for an Invasive Species, the Common Brushtail Possum (*Trichosurus vulpecula*), in an Urban Environment

**DOI:** 10.1371/journal.pone.0058422

**Published:** 2013-03-04

**Authors:** Amy L. Adams, Katharine J. M. Dickinson, Bruce C. Robertson, Yolanda van Heezik

**Affiliations:** 1 Department of Zoology, University of Otago, Dunedin, New Zealand; 2 Department of Botany, University of Otago, Dunedin, New Zealand; Australian Wildlife Conservancy, Australia

## Abstract

Invasive species are often favoured in fragmented, highly-modified, human-dominated landscapes such as urban areas. Because successful invasive urban adapters can occupy habitat that is quite different from that in their original range, effective management programmes for invasive species in urban areas require an understanding of distribution, habitat and resource requirements at a local scale that is tailored to the fine-scale heterogeneity typical of urban landscapes. The common brushtail possum (*Trichosurus vulpecula*) is one of New Zealand’s most destructive invasive pest species. As brushtail possums traditionally occupy forest habitat, control in New Zealand has focussed on rural and forest habitats, and forest fragments in cities. However, as successful urban adapters, possums may be occupying a wider range of habitats. Here we use site occupancy methods to determine the distribution of brushtail possums across five distinguishable urban habitat types during summer, which is when possums have the greatest impacts on breeding birds. We collected data on possum presence/absence and habitat characteristics, including possible sources of supplementary food (fruit trees, vegetable gardens, compost heaps), and the availability of forest fragments from 150 survey locations. Predictive distribution models constructed using the programme PRESENCE revealed that while occupancy rates were highest in forest fragments, possums were still present across a large proportion of residential habitat with occupancy decreasing as housing density increased and green cover decreased. The presence of supplementary food sources was important in predicting possum occupancy, which may reflect the high nutritional value of these food types. Additionally, occupancy decreased as the proportion of forest fragment decreased, indicating the importance of forest fragments in determining possum distribution. Control operations to protect native birds from possum predation in cities should include well-vegetated residential areas; these modified habitats not only support possums but provide a source for reinvasion of fragments.

## Introduction

Within and across environments the spatial distribution of animal species reflects the availability and distribution of species-specific resources and the ability of species to reach and exploit them [1], [2], [3], [4], [5], [6], [7]. Landscape structure determines habitat availability and connectivity between resources, influencing species distributions and occupancy of habitat patches [8], [9]. Landscape structure is especially significant in fragmented habitats where important resources and habitat patches become heterogeneously distributed across the environment [7], [10], [11], [12]. Fragmentation of landscapes results in an overall loss of natural habitat, both spatially and structurally, and species which are sensitive to urbanisation (‘urban avoiders’) [5] may be limited to small patches of natural habitat isolated within a matrix of modified habitats [9], [13]. Spatial isolation of patches can limit movements between patches by animals that are unable to disperse across the modified matrix [5], [8], [14]. Furthermore, edge effects can reduce the quality of the habitat within the patch with changes in microclimate, soil conditions, plant composition and species interactions [9], [10], [15]. The degree of fragmentation within a landscape and the nature of the matrix will influence the amount of time individuals spend outside of their preferred natural habitat, therefore altering their normal distribution patterns [10], [16]. The impacts of habitat fragmentation on animal distributions are particularly significant in urban landscapes, which are becoming increasingly prevalent as the world’s human population continues to grow [5], [17], [18]. In urban environments, the ecosystem has been transformed into hybrid systems consisting of fragments of original habitat that are often small and embedded within a matrix of highly-modified and human-dominated habitat [14].

Urban environments can also provide novel resources and habitats. Species that are sufficiently behaviourally and biologically flexible to exploit resources within modified matrix habitats are known as ‘urban adapters’ and are often generalist species [5], [17], [19]. For example, red foxes (*Vulpes vulpes*) exploit anthropogenic food sources and alter spatial behaviours to adapt to urban environments [20], [21]. Species such as raccoons (*Procyon lotor*), which require forest fragments, particularly for shelter, can also frequent and exploit resources in the urban matrix, such as vegetable gardens and garbage [5]. Invasive species, which tend to be opportunistic foragers and behaviourally flexible, are usually more successful at adapting to urban environments [22].

An invasive urban adapter in New Zealand is the common brushtail possum (*Trichosurus vulpecula*) which is a small to medium-sized generalist folivorous marsupial [23], [24]. Introduced into New Zealand from Australia in 1858, it has a wide distribution throughout both countries that includes urban environments [24], [25], [26]. In New Zealand, possums are an invasive pest species, primarily occupying forest habitats where they exist at densities far higher than in their native Australian range [24], [25], [27], [28], where densities are constrained by the palatability and nutritional content of forest vegetation [29], [30], [31]. Although primarily folivorous, possum distribution is also influenced by the distribution and availability of nutrient and energy-rich, non-foliar food resources (e.g. fruits and flowers) which are a sought-after supplementary food source [32], [33], [34], [35].

Little is known about the distribution of possums throughout urban habitats, but it is likely to be mainly influenced by the distribution of food sources [36]. In Australia, possums can exist at higher densities in urban areas [37], [38], [39] than in native forests, where they move between native remnants and adjacent residential areas, exploiting nutritionally valuable food sources in private gardens (e.g. cultivated garden plants, vegetable gardens, fruit trees, compost, food scraps), human-subsidised food left out intentionally and artificial shelter resources (e.g. roof awnings, floor spaces) [23], [28], [39], [40]. Although studies suggest that urban possums do not establish a home range independently of forest fragments [23], [28], [39], [40].

In contrast, New Zealand forests support possum densities comparable to those found in Australian urban areas due to the greater palatability and nutritional quality of the vegetation [29], [41]. Possums in New Zealand cause severe damage to native vegetation, they compete with native birds for resources and they eat birds and their eggs [32], [42]. They are therefore likely to have negative impacts on avian diversity and community structure in at least some components of the urban landscape. However, the highly palatable and productive nature of New Zealand forests [29] may mean that there is less incentive than in Australia for possums to move out of urban forest fragments to occupy other less densely vegetated habitats. It is important to determine the extent to which invasive adaptable species, such as brushtail possums, have established throughout the heterogeneous urban landscape to evaluate potential impacts on native biodiversity and to guide effective management strategies, which need to be based on a more comprehensive understanding of the distribution of the species across non-traditional habitats.

This study used site occupancy methodology to assess the distribution of invasive possums and determine environmental factors influencing their occupancy throughout an urban landscape. This research was conducted during summer when impacts on urban avian populations will be the greatest due to greater movements by possums [43] and the vulnerability of nesting adults, eggs and nestlings to possum predation. We evaluated occupancy across five urban habitat types: forest remnants, amenity parks and residential areas representing typical variation in garden size and structure. We predicted that possums would have the highest probability of occupancy within forest fragments due to the availability of their primary dietary item, plant foliage. Within residential areas, we predicted that possums would display higher occupancy in areas composed mainly of properties with large, well-established gardens, as well as in areas with supplementary food sources due to the higher nutritional and energy value of these non-foliar food items.

## Methods

### Ethics Statement

This study was conducted with permission from the Dunedin City Council to undertake research relating to common brushtail possums within the city’s parks and reserves and by householders to set-up WaxTags on private property.

### Study Area

We conducted field work within the city boundaries of Dunedin (∼120,000 inhabitants), New Zealand (45°52′S, 170°30′E). The 22,500 ha urban area included a green town belt featuring remnant forest fragments totalling 145 ha, stand-alone vegetation fragments, amenity parks and residential areas of differing levels of urbanisation characterised by housing density (Table 1). We defined five distinguishable urban habitat types from a GIS-based habitat map of Dunedin City [44]: forest fragments, amenity spaces, Residential 1 (Res 1), Residential 2 (Res 2) and Residential 3 (Res 3; see Table 1 for further details).

**Table 1:** Descriptions of the five urban habitat types in which common brushtail possums (*Trichosurus vulpecula*) were surveyed in Dunedin, New Zealand produced by Freeman and Buck [44].

Habitat Type	Habitat Description
Forest fragment	Structure-rich tree stands composed of both exotic and native tree species forming closed canopies ranging from one hectare to 24 hectares in area (mean = 4 hectares) which are surrounded by modified residential landscapes. Fragments have a similar distribution throughout Res 1 and Res 2 habitats but are largely absent from Res 3 habitat.
Amenity	Amenity spaces including council recreational parks or playgrounds, playing fields and golf courses in which grass is mown regularly with edges varying from houses and roads (N = 7), to bare edges with scattered trees and shrubs (N = 14), to completely enclosed by mature trees (N = 9).
Res 1	Residential areas with greater than one third of the property size comprised of mature, structurally-complex gardens containing an assortment of lawns, hedges, shrubs, and large established trees. Green cover totals 70% with a mean housing density of 11.6/ha (SD = 1.98, N = 14) [58].
Res 2	Residential areas with greater than one third of the property size comprised of structurally-less complex gardens dominated by lawns. Green cover ranges between 42–50% with a mean housing density of 12.52/ha (SD = 2.27, N = 20 suburbs) [58].
Res 3	Residential areas with no garden or where less than one third of the property is garden dominated by flowerbeds or lawn. Green cover totals 30% with a mean housing density of 28.6/ha (SD = 3.14, N = 6 suburbs) [58].

### Brushtail Possum Surveys

Occupancy data were collected by surveying thirty randomly generated locations in each of the five habitats (*N = *150) using ArcGIS 9.3.1 software [45] and the GIS-based habitat map of Dunedin City. We chose fifteen locations randomly from each habitat type to be sampled within one of two weekly survey periods over summer which coincided with favourable weather conditions (mostly fine with no heavy rain or strong winds) [46]: possums are most likely to be active, providing the highest detection probabilities, during fine weather. Weekly intervals were selected in accordance to the national standardised WaxTag protocol, as possums are more likely to be detected over a period of seven nights, during which time individuals should occupy any given part of their home range [47], [48].

Following the national protocol for WaxTag use, we detected possum presence by placing three peanut butter-flavoured WaxTags and a lure at each of the 150 sites and leaving them for seven consecutive nights [47], [48]. We nailed WaxTags 70 cm above the ground to a randomly chosen tree (or post in Res 3 gardens which lacked trees) and spaced these 10 m apart to enable independence [47], [49]. Contagion, an occurrence where an individual is detected at more than one sampling location due to actively seeking out the monitoring device for the bait [50], was prevented by the use of the above spacing and the unpalatable nature of the wax [48], [51]. The use of three independent WaxTags at each site acted as repeat surveys within the same visit [52]. Habitat characteristics, considered as likely influences on occupancy probability were recorded for each location. This included presence of supplementary food sources consisting of any type of fruit tree (fruiting and non-fruiting), vegetable gardens (presence was recorded if any vegetables were seen growing on the property) and un-covered compost heaps. Connectivity was assessed by calculating the percentage of area of forest fragments within a 500 m buffer mapped around each site, which reflected the average nightly movements of possums (A. Adams, unpubl. data) using ArcGIS 9.3.1 software [45].

### Statistical Analysis

We classified bite marks from WaxTags to animal species [53] using possum presence/absence data to generate naïve occupancy estimates (probability of occupancy excluding detection probability). Habitat occupancy (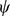) and detectability (*p*) were further investigated using a single-season analysis within the computer software PRESENCE version 3.0 which incorporates incomplete detectability, producing more reliable estimates [52], [54].

The initial analysis to investigate occupancy of possums within an urban environment used a simple spatially-explicit habitat model that assumed 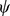 and *p* were constant (designated as “.” in models) over all sampling periods and habitat types, 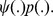 An alternative model including habitat type as a covariate, 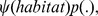
was then constructed.

To further investigate possum presence in an urban setting, we sub-sampled the presence/absence dataset to include only the three residential habitat types (Res 1, Res 2, Res 3; *N* = 90). This enabled us to examine possum occupancy in relation to the type of residential habitat and habitat characteristics (supplementary food items; area (%) of forest fragment within a buffer). We excluded forest and amenity habitats because these variables contained all zeros for supplementary food sources, which causes non-convergence in PRESENCE models. Models were compared based on the relative difference in Akaike’s second-order corrected Information Criterion (AICc) values corrected for small sample sizes [55]. We evaluated the fit of the best models using the threshold independent area under the curve (AUC) of Receiver Operating Characteristic (ROC) plots [56].

## Results

We detected brushtail possums at least once at 59 sites (N* = *150 sites) giving a naïve overall occupancy estimate of 0.39 across all habitats. Of the 30 survey locations for each habitat type, possums were detected at nearly all forest locations (28), just under half of the amenity (13) and Res 1 (12) locations dropping to 6 locations in Res 2 and 0 in Res 3 locations. Naïve occupancy estimates were 0.93 in forest, 0.43 in amenity space, 0.40 in Res 1, 0.20 in Res 2 and 0 in Res 3.

All three residential habitats had the same percentage (20%) of properties containing fruit trees, and about half of these within each habitat were fruiting during the sampling period. Proportions of properties containing vegetable gardens varied between residential habitat types: 30% of Res 1, 43% of Res 2 and 10% of Res 3 properties. Res 1 and Res 2 had a similar proportion of properties containing some form of compost: 27% and 23% respectively while only 0.7% of Res 3 properties had compost heaps.

When all habitat types were included, the model with the most support was where occupancy varied as a function of habitat type (Table 2). Occupancy of possums (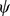) was highest in forest fragments, followed by amenity, Res 1, and Res 2 with lowest occupancy in Res 3 habitat (Table 2).

**Table 2:** Comparison of habitat models using Akaike’s second-order corrected Information Criterion (AICc) to obtain site occupancy (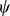
±1 SE) and detection probabilities (*p*±1 SE) for common brushtail possums (*Trichosurus vulpecula*) in five urban habitat types, Dunedin, New Zealand.

								*ψ*			
Model Description	AICc	Δ*i*	*w_i_*	Model Likelihood	K	Forest	Amenity	Res 1	Res 2	Res 3	*p*
psi(Habitat)p(.)	369.43	0.00	1.00	1.00	6	1.00 (0.00)	0.50 (0.11)	0.46 (0.10)	0.23 (0.08)	<0.0001 (0.00)	0.49 (0.05)
psi(.)p(.)	434.16	64.73	0.00	0.00	2	0.47 (0.05)	0.47 (0.05)	0.47 (0.05)	0.47 (0.05)	0.47 (0.05)	0.46 (0.05)

**Δ** *i* = AICc differences, *w_i_* = Akaike weights; K = number of parameters; (.) = constant probability.

When forest and amenity habitats were excluded, we fitted eight potential models varying in their combinations of covariates to the observed data (Table 3). Habitat type, supplementary food items and percentage area of forest fragment within a buffer all improved model fit (Table 3). In the top-ranking model, Res 1 had the highest occupancy estimate for possums followed by Res 2 then Res 3 habitats (Table 4). Therefore, possums are likely to be found in residential areas with structurally-complex vegetation, supplementary food items and greater proximity to forest fragments (Table 5).

**Table 3:** The model set investigating site occupancy for common brushtail possums (*Trichosurus vulpecula*) in five urban habitat types, Dunedin, New Zealand.

ModelDescription	AICc	Δ*i*	*w_i_*	ModelLikelihood	K	Deviance
psi(Residential+Food+Area),p(.)	146.93	0.00	0.63	1.0000	6	133.92
psi(Residential),p(.)	147.96	1.03	0.37	0.5979	4	139.49
psi(.),p(.)	163.30	16.37	0.00	0.0003	2	159.16
psi(Area),p(.)	164.95	18.02	0.00	0.0001	3	158.67
psi(Food),p(.)	169.51	22.58	0.00	0.0000	2	165.37
psi(Food+Area),p(.)	171.61	24.68	0.00	0.0000	3	165.33
psi(Residential+Area),p(.)	173.24	26.31	0.00	0.0000	5	162.53
psi(Residential+Food),p(.)	173.77	26.84	0.00	0.0000	5	163.06

**Δ** *i* = AICc differences, *w_i_* = Akaike weights; K = number of parameters; (.) = constant probability.

**Table 4:** Proportions (±1 SE) and amount of area occupied by common brushtail possums (*Trichosurus vulpecula*) for the two model sets across different urban habitats, Dunedin, New Zealand.

Analysis	Habitat Type	Area (ha)	Proportion ofOccupied Area ( 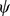 )	Absolute amounts ofOccupied Area (ha)	Detectability (*p*)
All Habitats	Forest	145	1.00 (0.00)	145.0	0.49 (0.05)
	Amenity	919	0.50 (0.11)	459.5	0.49 (0.05)
	Res 1	302	0.46 (0.10)	138.9	0.49 (0.05)
	Res 2	1921	0.23 (0.08)	441.8	0.49 (0.05)
	Res 3	381	0.00 (0.00)	0.0	0.49 (0.05)
**Total**		3668		1185.2	
Residential Habitats	Res 1	302	0.45 (0.14)	135.9	0.44 (0.08)
	Res 2	1921	0.26 (0.18)	499.5	0.44 (0.08)
	Res 3	381	0.00 (0.00)	0.0	0.44 (0.08)
**Total**		2604		635.4	

**Table 5:** Variables included in the final model for occupancy probability across the residential habitat types, their coefficients (β) and standard errors (S.E.).

Variable	β	S.E.
Res I	40.68	1.46
Res II	39.17	1.55
Res III	−41.32	1.46
Food	1.64	0.74
% Area Forest	36.37	23.39

We then determined the current level of occupancy of common brushtail possums throughout the Dunedin urban environment using the estimates generated from the top models (Table 4). The modelling predicted that all urban forest habitat is occupied by brushtail possums, while about half of the amenity and Res 1 habitats will have possums present (Table 3). Presence declined in Res 2 habitats, but due to the size of this habitat category, a considerable area was still estimated to be occupied by brushtail possums (499.5 ha).

The predictive ability of the best models both showed moderately good accuracy. The 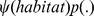 model examining possum presence across all five urban habitat types had an AUC of 0.84±0.03 indicating that the model could correctly discriminate between the presence and true absence of possums 84% of the time, while the 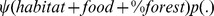 model investigating occupancy of possums across residential habitat types had an AUC of 0.77±0.05.

## Discussion

Habitat occupancy of the common brushtail possum in the urban Dunedin landscape during summer reflected the heterogeneous distribution of resources and patch connectivity, emphasising the influence that landscape structure and resulting resource availability have on the distribution of this species [57]. Highest occupancy rates in natural habitats (nearly all forest fragments sampled were occupied) reflect the traditional distribution of brushtail possums throughout forests, which contain important resources including den sites and their primary dietary item, plant material, which makes up 50–90% of their total diet [24], [33].

Possum occupancy declined outside of forest fragments, decreasing with increased modification of matrix habitat. Amenity habitat had the second highest occupancy estimate which is most likely explained by the surrounding characteristics of this open, green space. Brushtail possums were detected in seven of the nine amenity locations that were completely enclosed by complex vegetation, but in only three of seven amenity locations surrounded by housing and roads and three of fourteen locations that had bare edges with a scattering of trees or shrubs. The presence of mature, complex vegetation around amenity space is likely acting as an available source of food and shelter. Urban parks in Australia containing trees also have associated high possum densities which are thought to be related to foraging opportunities from both surrounding vegetation and human food scraps [36]. The behavioural flexibility of the invasive brushtail possum enables individuals to exploit both novel food sources (fruit trees, food scraps, garden plants including their flowers and tree foliage) and novel den sites (trees, wood piles, roofs, chimneys, spaces in and under buildings) available in private residential gardens [23], [28]. However, our results indicate their distribution across residential areas is constrained by the amount and structure of the vegetation. Within residential areas, possums were most likely to occur in areas with large gardens situated close to forest fragments, containing structurally-complex vegetation, and some source of supplementary food. Res 1 and 2 habitats have similar-sized gardens, but they vary in vegetation characteristics, with resources becoming patchier in Res 2 habitats due to the lower proportion of vegetation cover and the simpler nature of the gardens [58]. This was reflected in lower occupancy rates: 23% of res 2 habitat compared to 46% in res 1 habitat.

Brushtail possums residing in urban bush fragments in Australia also frequent gardens of surrounding residential properties which contain a high diversity of exotic plant species, including shrubs, trees and their fruits and flowers. These are of higher nutritional value and less toxic than *Eucalyptus* species [59], which constitute a major part of brushtail possum diet in their native range [24], [31], [40]. Native forest species in New Zealand typically have greater nutritional and energy value than native vegetation in Australia and they lack chemical defence mechanisms [29], [60]. Nevertheless, even though forest fragments in urban New Zealand consist of vegetation that is more palatable and nutritionally valuable than native vegetation fragments in Australia [41], possums in New Zealand still used residential gardens to supplement their diet with highly nutritious and energy rich food items.

The presence of fruit trees primarily, but also vegetable gardens and compost heaps within residential areas, all influenced brushtail possum presence. The presence of fruits in other New Zealand environments are known to influence the distribution of brushtail possums: seasonal fruiting in forested environments results in an increase in the number of possums eating often large quantities of fruit which can account for up to 42% of their diet [32], [61], [62], while possums in rural landscapes are known to travel up to 1600 m to reach preferred dietary items, such as apples [43], [63], [64]. Fruit, vegetables and compost heaps all act as a supplementary food source, providing possums with dietary items high in energy and nutrients to help compensate for their limited ability to extract enough nutrients and energy from foliage alone [23], [33]. Even though the proportions of these supplementary food sources were similar across Res 1 and Res 2 habitats, occupancy rates were quite different, suggesting that general habitat characteristics and levels of housing density and green cover are more important in determining occupancy. Despite appropriate food supplies being available in Res 3 habitat, albeit at lower densities, these areas may remain unoccupied because they are situated far from forest fragments and are therefore not well connected to possum’s traditional habitat. The virtual absence of structurally-complex vegetation used for both food and den sites in Res 3 habitat may also be a limiting factor.

Res 1 and 2 habitats were typically located in suburbs in which possums had shorter distances to travel from forest fragments to access food resources in gardens. Low density housing with private gardens containing greater plant diversity are often characteristics found in high socio-economic neighbourhoods (Y. van Heezik, unpubl. data), which also tend to be located close to public green spaces [65], [66]. This clustering of vegetatively diverse gardens close to forest fragments may facilitate the movement of possums between forest fragments and residential habitats. Similar trends have been documented in other urban areas with Baker & Harris [67] showing that the use of private gardens by mammalian species within residential areas of Great Britain decreased as urbanisation increased due to smaller garden sizes, increased human disturbance and increased distance to natural or semi-natural areas. The degree of connectivity between habitat patches and the presence of preferred habitat has been shown to significantly affect species occupancy of the landscape by affecting dispersal across less preferred habitat types [8], [68].

Occupancy of possums within urban habitats, especially those with high housing densities, may be further limited by a number of factors: predation from introduced mammals [69], [70], in this case dogs, increased noise (i.e. children, pets and traffic) [71], and pollutants [69]. While possums were not detected in Res 3 habitat, it cannot be concluded that they were absent. Species may occupy habitats in low densities, resulting in very low probabilities of being detected using the detection method employed during the sampling period [72].

The naïve site occupancy estimates underestimated the probability of occupancy by 3–7%, as imperfect detectability of the species was not accounted for [52], [73]. However, because detectability of a species at a site can be influenced by numerous factors including sampling effort (including type and timing of the survey), weather conditions, animal behaviour, local density of the species, random chance and observer experience [74], [75], [76], it is important to incorporate the probability of detection into data analysis to avoid misleading, inaccurate conclusions both of occupancy rates and effects of variables [52], [76].

Effective management of invasive species requires knowledge of its distribution throughout a landscape to indicate where control should be focussed and the intensity of control required. We have shown that in a New Zealand urban landscape, despite the high nutritional quality of fragment vegetation, brushtail possums still occur across residential areas that support structurally complex vegetation. These vegetation characteristics are also associated with higher native bird diversity and abundance [77], indicating that negative impacts of possums on birds will extend across large parts of the city. While invasive species management can become less cost-effective in habitats where their probability of occurrence is low [78], community involvement in possum surveillance and control is an option in residential areas that is already being carried out in some areas in New Zealand, and has the potential to achieve biodiversity gains.
